# How malaria models relate temperature to malaria transmission

**DOI:** 10.1186/1756-3305-6-20

**Published:** 2013-01-18

**Authors:** Torleif Markussen Lunde, Mohamed Nabie Bayoh, Bernt Lindtjørn

**Affiliations:** 1 Bjerknes Centre for Climate Research, University of Bergen, Norway; 2 Centre for International Health, University of Bergen, Norway; 3 KEMRI/CDC Research and Public Health Collaboration, Kisumu, Kenya; 4 Bjerknes Centre for Climate Research, Uni Research, Norway

**Keywords:** *Anopheles gambiae* sensu stricto, Climate, Temperature, Mathematical model

## Abstract

**Background:**

It is well known that temperature has a major influence on the transmission of malaria parasites to their hosts. However, mathematical models do not always agree about the way in which temperature affects malaria transmission.

**Methods:**

In this study, we compared six temperature dependent mortality models for the malaria vector *Anopheles gambiae* sensu stricto. The evaluation is based on a comparison between the models, and observations from semi-field and laboratory settings.

**Results:**

Our results show how different mortality calculations can influence the predicted dynamics of malaria transmission.

**Conclusions:**

With global warming a reality, the projected changes in malaria transmission will depend on which mortality model is used to make such predictions.

## Background

Since the 1950s, near-surface global temperatures have increased by about 0.5-0.6°*C*[1], and it is likely that temperatures will continue to increase over the next century [2]. Model predictions, reported widely in climate policy debates, project that a warmer climate could increase malaria caused by the parasites *Plasmodium falciparum* and *P. vivax* in parts of Africa [3]. Malaria is transmitted by mosquitoes of the *Anopheles* genus, with *Anopheles gambiae s.s.*, *An. arabiensis* and *An. funestus* being the dominant vector species in Africa [4,5].

These projections rely on knowledge about how the malaria parasite and anopheline vectors respond to changes in temperature. While a lot is known [6] about how parasite development is influenced by temperature [7], the same cannot be said for mosquitoes. In addition to temperature, humidity [8,9], breeding site formation [10], and competition between mosquitoes [11,12] are important factors controlling the number of vectors at any time.

Climate predictions about humidity and precipitation are more uncertain than temperature projections. Therefore, it is of interest to see if a consensus exists between different malaria models about how temperature alone influences malaria transmission. In the past, studies have suggested that the optimal temperature for malaria transmission is between 30 and 33°*C*[13-15].

Here, we compare six mortality models (Martens 1, Martens 2, Bayoh-Ermert, Bayoh-Parham, Bayoh-Mordecai and Bayoh-Lunde) to reference data (control) for *Anopheles gambiae s.s.*, and show how these models can alter the expected consequences of higher temperatures. The main purpose of the study is to show if there are any discrepancies between the models, with consequences for the ability of projecting the impact of temperature changes on malaria transmission.

We have focused on models that have been designed to be used on a whole continent scale, rather than those that focus on local malaria transmission [10,16,17].

## Methods

### Survival models

Six different parametrization schemes have been developed to describe the mortality rates for adult *An. gambiae s.s.*. These schemes are important for estimating the temperature at which malaria transmission is most efficient. The models can also be used as tools to describe the dynamics of malaria transmission. In all of the equations presented in this paper, temperature, *T* and *T*_*air*_are in °*C*.

#### Martens 1

The first model, which is called Martens scheme 1 in Ermert *et al.*[18], and described by Martens *et al.*[19-21], is derived from three points, and shows the relationship between daily survival probability (*p*) and temperature (*T*). This is a second order polynomial, and is, mathematically, the simplest of the models.

(1) $$p(T)=-0.0016\cdot T^{2}+0.054\cdot T+0.45$$

#### Martens 2

In 1997 Martens [21] described a new temperature-dependent function of daily survival probability. This model has been used in several studies [13,14,22,23]. In the subsequent text this model is named Martens 2. Numerically, this is a more complex model than Martens 1, and it increases the daily survival probability at higher temperatures.

(2) $$p(T)=e^{-\frac{1}{-4.4+1.31\cdot T-.03\cdot T^{2}}}$$

#### Bayoh-Ermert

In 2001, Bayoh carried out an experiment where the survival of *An. gambiae s.s.* under different temperatures (5 to 40 in 5°*C* steps) and relative humidities (RHs) (40 to 100 in 20% steps) was investigated [24]. This study formed the basis for three new parametrization schemes. In the naming of these models, we have included Bayoh, who conducted the laboratory study, followed by the author who derived the survival curves.

In 2011, Ermert *et al.*[18] formulated an expression for *Anopheles* survival probability; however, RH was not included in this model. In the text hereafter, we name this model Bayoh-Ermert. This model is a fifth order polynomial.

Overall, this model has higher survival probabilities at all of the set temperatures compared with the models created by Martens.

(3) $$\begin{array}{l} p(T)=-2.123\cdot 10^{-7}\cdot T^{5}+1.951\cdot 10^{-5}\cdot T^{4}\\ \;-6.394\cdot 10^{-4}\cdot T^{3}+8.217\cdot^{-3}\cdot T^{2}\\ \;-1.865\cdot 10^{-2}\cdot T+7.238\cdot 10^{-1} \end{array}$$

#### Bayoh-Parham

In 2012, Parham *et al.*[25] (designated Bayoh-Parham in subsequent text) included the effects of relative humidity and parametrized survival probability using the expression shown below. This model shares many of the same characteristics as the Bayoh-Ermert model. The mathematical formulation is similar to the Martens 2 model, but constants are replaced by three terms related to RH (*β*_0_*β*_1_*β*_2_).

(4) $$p(T,\text{RH})=e^{-{\left(T^{2}\cdot \beta_{2}+T\cdot \beta_{1}+\beta_{0}\right)}^{-1}}$$

where *β*_0_=0.00113·*RH*^2^−0.158·*RH*−6.61, *β*_1_=−2.32·10^−4^·*RH*^2^ + 0.0515·*RH* + 1.06, and *β*_2_=4·10^−6^·*RH*^2^−1.09·10^−3^·*RH*−0.0255.

For all models reporting survival probability, we can rewrite *p* to mortality rates, *β*according to:

(5) β=−ln(p)

#### Bayoh-Mordecai

Recently, Mordecai *et al.*[26] re-calibrated the Martens 1 model by fitting an exponential survival function to a subset of the data from Bayoh and Lindsay [24]. They used the survival data from the first day of the experiment and one day before the fraction alive was 0.01. Six data points were used for each temperature.

(6) $$p(T)=-0.000828\cdot T^{2}+0.0367\cdot T+0.522$$

#### Bayoh-Lunde

From the same data [24], Lunde *et al.*[27], derived an age-dependent mortality model that is dependent on temperature, RH, and mosquito size. This model assumes non-exponential mortality as observed in laboratory settings [24], semi-field conditions [28], and in the field [29]. In the subsequent text we call this model Bayoh-Lunde. The four other models use the daily survival probability as the measure, and assume that the daily survival probability is independent of mosquito age. The present model calculates a survival curve (*ϖ*) with respect to mosquito age. Like the Bayoh-Parham model, we have also varied the mosquito mortality rates according to temperature and RH.

Because mosquito size is also known to influence mortality [8,9,30,31], we applied a simple linear correction term to account for this. In this model, the effect of size is minor compared with temperature and relative humidity. The survival curve, *ϖ*, is dependent on a shape and scale parameter in a similar manner as for the probability density functions. The scale of the survival function is dependent on temperature, RH, and mosquito size, while the scale parameter is fixed in this paper.

The mortality rate, *β*_*n*_(*T*,*RH*,*size*) (equation 7) is fully described in Additional file 1, with illustrations in Additional files 2, and 3.

(7) $$\begin{array}{c} \beta_{n}(T,\text{RH},\text{size})=\frac{\ln\left(\frac{\varpi_{N,m_{t_{2}}}}{\varpi_{N,m_{t_{1}}}}\right)}{\mathrm{\Delta t}} \end{array}$$

### Biting rate and extrinsic incubation period

The equations used for the biting rate, *G*(*T*), and the inverse of the extrinsic incubation period (EIP, *pf*) are described in Lunde et al. [27]. For convenience, these equations and their explanations are provided in Additional file 1. The extrinsic incubation period was derived using data from MacDonald [7], while the biting rate is a mixture of the degree day model by Hoshen and Morse [32], and a model by Lunde *et al.*[27]. Since our main interest in this research was to examine how mosquito mortality is related to temperature in models, we used the same equation for the gonotrophic cycle for all of the mortality models. If we had used different temperature-dependent gonotrophic cycle estimates for the five models, we would not have been able to investigate the effect of the mortality curves alone.

### Malaria transmission

We set up a system of ordinary differential equations (ODEs) to investigate how malaria parasites are transmitted to mosquitoes. Four of the mortality models (equations 1, 2, 3, and 4) are used in a simple compartment model that includes susceptible (*S*), infected (*E*) and infectious mosquitoes (*I*) (equation 8):

(8) $$\begin{array}{ll} \frac{\text{dS}}{\text{dt}} & =-(\beta +G(T)\cdot H_{i})\cdot S\\ \frac{\text{dE}}{\text{dt}} & =(G(T)\cdot H_{i})\cdot S-(\beta +\text{pf})\cdot E\\ \frac{\text{dI}}{\text{dt}} & =\text{pf}\cdot E-\beta \cdot I \end{array}$$

where *H*_*i*_is the fraction of infectious humans, which was set to 0.01. *G*(*T*) is the biting rate, and *pf* is the rate at which sporozoites develop in the mosquitoes. The model is initialized with *S*=1000, *E*=*I*=0 and integrated for 150 days with a time step of 0.5. As the equations show, there are no births in the population, and the fraction of infectious humans is held constant during the course of the integration. This set-up ensures that any confounding factors are minimized, and that the results can be attributed to the mortality model alone.

Because the Lunde *et al.*[27] (Bayoh-Lunde) mortality model also includes an age dimension, the differential equations must be written taking this into account. Note that the model also can be used in equation 8 if we allow *β* to vary with time.

We separate susceptible (*S*), infected (*E*) and infectious (*I*), and the subscript denotes the age group. In total there are 25 differential equations, but where the equations are similar, the subscript *n* has been used to indicate the age group.

Formulating the equation this way means we can estimate mosquito mortality for a specific age group. We have assumed that mosquito biting behaviour is independent of mosquito age; this formulation is, therefore, comparable to the framework used for the exponential mortality models.

The number of infectious mosquitoes is the sum of *I*_*n*_, where *n*=2,…,9.

(9) $$\begin{array}{l} \frac{dS_{1}}{\text{dt}}=-(\beta_{1}+a_{1})\cdot S_{1}\\ \frac{dS_{n}}{\text{dt}}=a_{n-1}\cdot S_{1}-(\beta_{n}+a_{n}+G(T)\cdot H_{i})\cdot S_{n}\\ \;n=2,3,\mathrm{..},9\\ \frac{dS_{n}}{\text{dt}}=G(T)\cdot H_{i}\cdot S_{2}-(\beta_{2}+a_{2}+\text{pf})\cdot E_{2}\\ \frac{dE_{n}}{\text{dt}}=G(T)\cdot H_{i}\cdot S_{n}+a_{n-1}\cdot E_{n-1}\\ \;-(\beta_{n}+a_{n}+\text{pf})\cdot E_{n}\\ \;n=3,4,\mathrm{..},9\\ \frac{dI_{2}}{\text{dt}}=\text{pf}\cdot E_{2}-(\beta_{2}+a_{2})\cdot I_{2}\\ \frac{dI_{n}}{\text{dt}}=\text{pf}\cdot E_{n}+a_{2}\cdot I_{n-1}-(\beta_{n}+a_{n})\cdot I_{n}\\ \;n=3,4,\mathrm{..},9 \end{array}$$

Age groups for mosquitoes (*m*) in this model are *m*_1_=[0,1], *m*_2_=(2,4], *m*_3_=(5,8], *m*_4_=(9,13], *m*_5_=(14,19], *m*_6_=(20,26], *m*_7_=(27,34], *m*_8_=(35,43], *m*_9_=(44,*∞*] days, and coefficients *a*_*n*_, where *n*=1,2,…,9, are 1.000, 0.500, 0.333, 0.250, 0.200, 0.167, 0.143, 0.125, 0.067. The rationale behind these age groups is that as mosquitoes become older, there is a greater tendency of exponential mortality compared to younger mosquitoes.

This model has initial conditions *S*_1_=1000, and all other 0.

A note on the use of ODEs and rate calculations can be found in Additional file 4.

### Validation data

To validate the models, we used the most extensive data set available on mosquito survival [24] under different temperatures (5 to 40 by 5°*C*) and RHs (40 to 100 by 20%) [24]; it is the same data that the Bayoh-Ermert, Bayoh-Parham and Bayoh-Lunde models were derived from. These data describe the fraction of live mosquitoes (*f*_*a*_) at time *t*, which allows us to validate the models over a range of temperatures. Because three of the models used the Bayoh and Lindsay data to develop the survival curves, this comparison is unrealistic for Martens models.

Hence, to account for this we have used three independent data sets to validate the fraction of infectious mosquitoes and the mosquito survival curves.

Scholte *et al.* (Figure two in [33]) published a similar data set, but this was based on a temperature of 27±1°*C* and a RH of 80±5*%*, whereas Afrane *et al.* (Figure two in [28]) used mean temperatures of 21.5 to 25.0 and RHs of 40-80%. Use of these data sets will allow us to complement the validation to determine if the patterns of malaria transmission are consistent with that of the control (Table 1). In addition to the data from Scholte *et al.*[33], we also found the following data set, which is suitable for validation of the survival curves but not the transmission process itself, because the data does not show the survival curve until all of the mosquitoes are dead [Kikankie, Master’s thesis (Figures three to eight, chapter 3, 25°*C*, 80% RH) [34]]. These results are also shown in Table 1. The additional validation only gives information about the model quality between 21, and 27°*C*; however, it serves as an independent model evaluation to determine if the results are consistent and independent of the data set used to validate the models.

**Table 1:** Skill scores

	**Control**	** AIC Control**	**Scholte**	**AF**	**BL mortality model**	**SK mortality model**
Martens 1	0.01	76 (56, 96)	0.00	0.03	0.36	0.25
Martens 2	0.38	9 (-14, 30)	0.55	0.37	0.54	0.45
Martens 3	0.65	-38 (-75, -9)	0.53	0.77	0.65	0.52
Bayoh-Ermert	0.27	30 (1, 58)	0.16	0.43	0.79	0.56
Bayoh-Parham	0.16	26 (-11, 55)	0.05	0.31	0.79	0.59
Bayoh-Lunde	0.90	-111 (-148, -81)	0.83	0.94	0.90	0.81
Bayoh-Mordecai	0.62	-53 (-82, -29)	0.58	0.70	0.57	0.49

Skill scores as defined in equation 11. “Control” represents the validation of infectious mosquitoes using the data from Bayoh and Lindsay [24], “Scholte” [33] represents the validation of infectious mosquitoes using the data from Scholte, “AF” represents the validation of infectious mosquitoes using the data from Afrane, “BL mortality model” represents the validation of the mortality model using the data from Bayoh and Lindsay [24], and “SK mortality model” represents the validation of the mortality model using data from Scholte [33] and Kikankie [34]. “AIC control” is Akaike information Criterion for “Control” (95 % confidence interval).

Using the data from Bayoh and Lindsay, Afrane *et al.* or Scholte *et al.*[33], we can calculate the fraction of mosquitoes that would become infectious at time *t*, using equation 8. We replace *β* with the time-dependent *β*(*t*), which is a time varying mortality rate. This approach was used for the data from [24] and [33].

(10) $$\beta (t)=-\text{ln}\left(\frac{f_{a}\left(t+\frac{1}{2}\right)}{f_{a}\left(t-\frac{1}{2}\right)}\right)$$

*β*(*t*) is linearly interpolated at times with no data. The reference data from Bayoh and Lindsay [24] are hereafter designated as the control data in the subsequent text, whereas data from Scholte *et al.*[33] is called Scholte in Table 1. Table 1 also shows the skill scores of the mortality model alone (for the figures in Additional file 3).

Because some of the schemes do not include RH, we have displayed the mean number of infectious mosquitoes, *I*, for schemes that do include it. For the validation statistics, RH has been included. However, for schemes where the RH has not been taken into account, single realization at all humidities has been employed.

### Validation statistics

Skill scores (*S*) are calculated following Taylor [35]:

(11) $$S_{s}=\frac{4\cdot {\left(1+r\right)}^{4}}{{\left({\hat{\sigma }}_{f}+1/{\hat{\sigma }}_{f}\right)}^{2}\cdot {\left(1+r_{0}\right)}^{4}}$$

where *r* is the Pearson correlation coefficient, *r*_0_=1 is the reference correlation coefficient, and ${\hat{\sigma }}_{f}$ is the variance of the control over the standard deviation of the model (*σ*_*f*_/*σ*_*r*_). This skill score will increase as a correlation increases, as well as increasing as the variance of the model approaches the variance of the model.

The Taylor diagram used to visualize the skill score takes into account the correlation (curved axis), ability to represent the variance (x and y axis), and the root mean square.

Another important aspect is determining at which temperatures transmission is most efficient. If mosquitoes have a peak of infectiousness at, for example, 20°*C* in one model, temperatures above this will lead to a smaller fraction of mosquitoes becoming infectious. A different model might set this peak at 27°*C*, so that at temperatures from 20-27°*C*, the fraction of infectious mosquitoes will increase, followed by a decrease at higher temperatures. Isolating the point at which the mosquitoes are the most efficient vectors for malaria parasites is important for assessing the potential impacts of climate change. To show the differences between the models, we report the temperature where the maximum efficiency for producing infectious mosquitoes was observed. This can be done by maximizing equation 12.

(12) $$\arg\underset{T\in [10,40]}{\text{max}}\int_{t=0}^{\infty }\text{Idt}$$

For the transmission process we also report Akaike information criterion (AIC) [36] from a generalized linear model with normal distribution. Since the observations are not independent, and residuals do not follow a normal distribution, we sample 100 values from the simulations 1000 times. We set the probability of sampling *y*_*i*,*j*_ equal to normalized (sum = 1) fraction of infected mosquitoes of the control. This method allow us to generate a model with normally distributed, non-correlated errors. Median AIC, with 95% confidence intervals are reported in Table 1.

## Results

**Figure 1:**
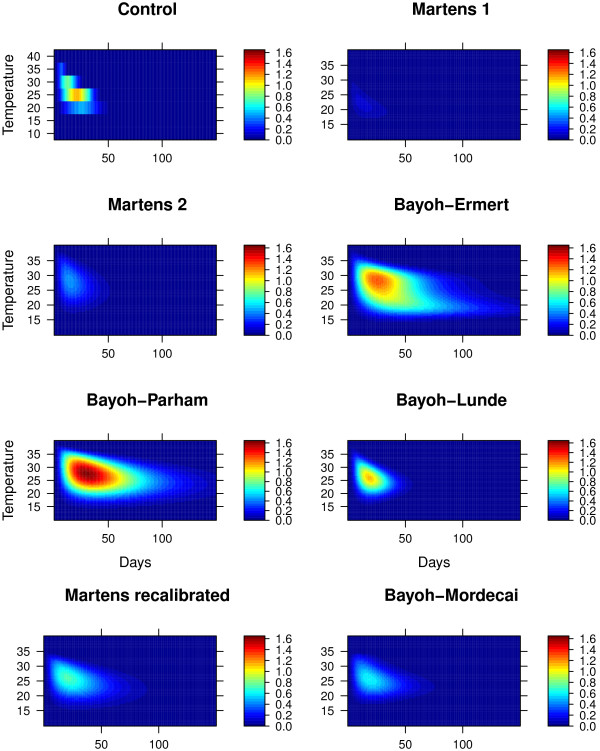
The percentage of infectious mosquitoes over time and temperature.

Figure 1 shows the percentage of infectious mosquitoes plotted against time (days) (x) and temperature (y). The control shows that the most efficient transmission occurs at 25°*C*, while the maximum percentage of infectious mosquitoes at any time is 1.1. We found that the Martens 1 and 2 models both underestimate the fraction of infectious mosquitoes, while the Bayoh-Ermert and Bayoh-Lunde models had comparable values. While the Bayoh-Parham model affords similar values at 40% RH, it overestimates the fraction of infectious mosquitoes at higher RHs (Additional file 3). There are also substantial differences at which the temperatures for transmission are most efficient.

**Figure 2:**
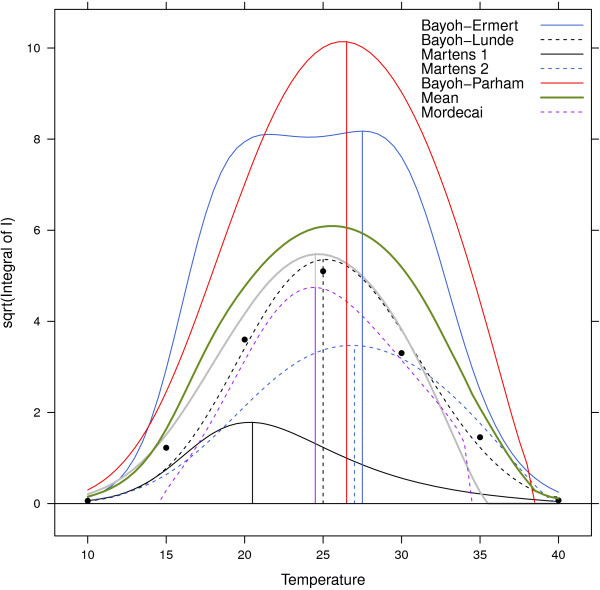
**Integral of infectious mosquitoes over temperature.** Models: Bayoh-Ermert (blue solid line), Martens 1 (black solid line), Martens 2 (blue dashed line), Martens 3 (grey solid line), Bayoh-Parham (red solid line), Lunde (black dashed line), and the mean value of the five models (green thick solid line). Black dots indicate the results for the control, and vertical lines show the temperature at which the maximum can be found (equation 12).

While Martens 1 has the most efficient transmission at 20.4°C, Martens 2 and Bayoh-Ermert show the transmission efficiency peaking at 26.8 and 27.5°*C*. Both the control and Bayoh-Lunde models peak at 25°*C*, as measured according to equation 12, Bayoh-Parham peaks at 26.3°*C*, and Bayoh-Mordecai peaks at 24.4°*C* (Figure 2).

The numerical solution of the Bayoh-Ermert mortality model also reveals that it has problems related to enhanced mosquito longevity at all of the selected temperatures; this effect was especially pronounced around 20°*C*. We also found that the Bayoh-Parham model has issues with prolonged mosquito survival.

**Figure 3:**
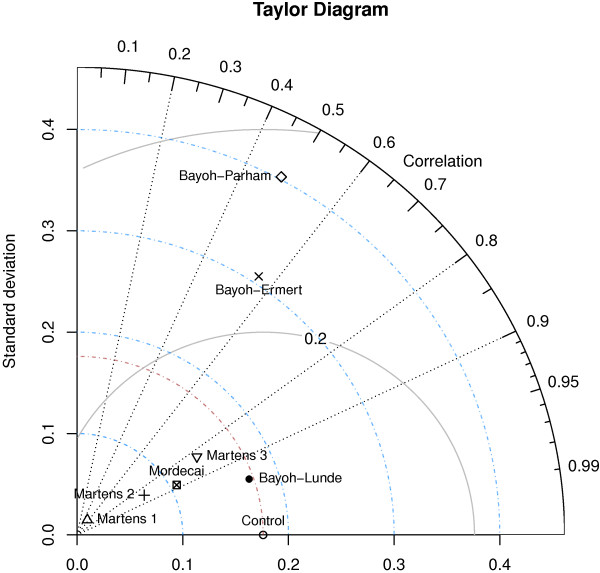
**Taylor diagram.** The model names are indicated next to the symbols. The x and y axes represent the standard deviations, the curved grey lines are the root mean square, while the dashed lines represent the Pearson correlation coefficient.

To evaluate the skill of the models, with emphasis on spatial patterns and variance, we investigated the skill score that was defined in equation 11. The standard deviation, root mean square and correlation coefficient are summarized in a Taylor diagram (Figure 3). Skill scores closer to 1 are a sign of better performance from a model (Table 1).

When validating the transmission process using the data from Bayoh and Lindsay (Table 1, column 1), the majority of the penalty for the Martens 1 and 2 models was due to the low variance, indicating that the mortality is set too high compared with the reference. Further analysis found that the Bayoh-Ermert model correlated poorly with the reference, and the variance, ${\hat{\sigma }}_{f}$, was too high. The Bayoh-Parham model also suffered from low correlation, as well as too high variance. Overall, the Bayoh-Lunde model has the highest skill score, followed by the Bayoh-Mordecai model. The patterns are consistently independent of the data used to validate the models with respect to the malaria transmission process. Validation of the survival curves alone, and their relationship with the transmission process, is discussed in the next section.

The relatively simple Martens 2 model ranked third among the models. We re-calibrated [37,38] the model using the data from Bayoh and Lindsay. The re-calibrated model (equation 13) generated a skill score of 0.65 (for the transmission process). In addition, Martens 2 was most efficient at 24.5°*C*. The Martens 3 model can be used for temperatures between 5 and 35°*C*.

(13) $$p(T)=e^{-\frac{1}{-4.31564+2.19646\cdot T-0.058276\cdot T^{2}}}$$

The newly calibrated Martens 2 model (hereafter called Martens 3), can be seen in Figure 2; the skill scores are reported in Table 1.

**Figure 4:**
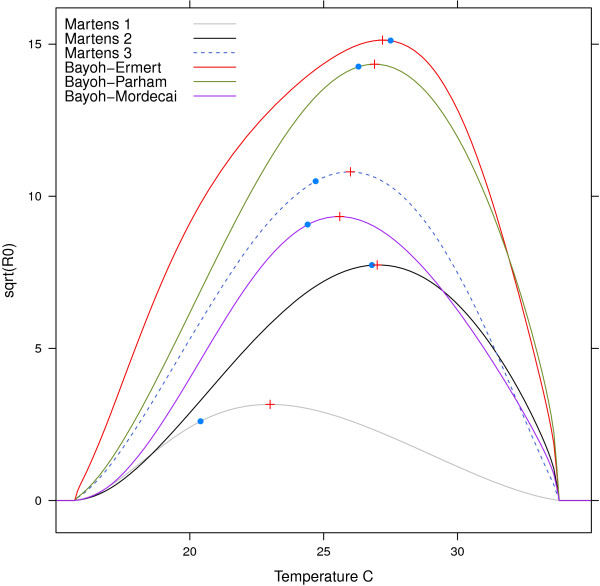
***R***_***0 ***_**as a function of temperature calculated using equation 2 in Mordecai *et al. * [**[26]**], but with different mortality models.** Blue dots represent optimal temperatures using the methods in this paper, and red crosses is the optimal temperature using the methods from Mordecai *et al.*[26].

To investigate how sensitive the results of the Mordecai *et al.*[26] analysis are to the choice of mortality model, we calculated the optimal temperature for malaria transmission using their full temperature-sensitive malaria *R*_0_ model (equation 2 in [26]). The mortality rate, *μ*(*T*), was replaced with −*ln*(*p*(*T*)) from the exponential models. Population density (*N*), and recovery rate, *r*, were set to 1, since these do not influence the optimal temperature for malaria transmission. The results can be seen in Table 2. Relative differences between the two methods is in the range from 1–11% (Table 2). Figure 4 shows *R*_0_ according to temperature (with *N*=1,*r*=1) for the exponential models. The maximum *R*_0_ranges from 10 (Martens 1) to 206 (Bayoh-Parham).

**Table 2:** Optimal temperature for malaria transmission

	**This paper**	***R***_**0**_** from Mordecai**	**Relative**
		***et al.***	**difference %**
Martens 1	20.4	23.0	11.98
Martens 2	26.8	27.0	0.74
Martens 3	24.7	26.0	5.13
Bayoh-Ermert	27.5	27.2	1.10
Bayoh-Parham	26.3	26.9	2.26
Bayoh-Lunde	25.2		
Bayoh-Mordecai	24.4	25.6	4.80

Optimal temperature calculated using the methods in this paper, and by using methodology in Mordecai *et al.*[26].

## Discussion and conclusions

The relationship between sporozoite development and the survival of infectious mosquitoes at different temperatures is poorly understood; therefore, any model projections relating the two should be interpreted with care. The Martens 2 and Bayoh-Ermert models suggest that areas of the world where temperatures approach 27°*C* could experience more malaria. Martens 3, Bayoh-Mordecai, and our model (Bayoh-Lunde) suggests that transmission is most efficient at around 25°*C*. The Martens 1 model peaks at 20.4°*C*, and Bayoh-Parham at 26.3°*C* (Figure 1). None of the models, except Bayoh-Lunde, capture all of the characteristics of the reference data, however.

Table 1 also shows the skill score for the mortality model alone. Both the Bayoh-Parham and the Bayoh-Ermert models have good representations of the survival curves. However, the nature of the exponential mortality curves gives them the choice of rapid mortality giving a reasonable, but underestimated, transmission process (Martens 2), or a good fit to the survival curves, which in turn makes the mosquitoes live too long, resulting in a poor transmission process (Bayoh-Parham and Bayoh-Ermert). Because the Bayoh-Lunde model offers a fair description of the survival curves as well as an age structure in the differential equations, we consider that the transmission process is well described. The Martens 1 and 2, Bayoh-Ermert, Bayoh-Mordecai and Bayoh-Parham models all assume constant mortality rates with age, and would, therefore, not benefit from being solved in an age-structured framework.

The Martens 1 model has been used in several studies [19-21], with the latest appearance by Gething *et al.* in this journal [39]. Considering the poor skill of the Martens 1 model, the validity, or etiology, of results presented in these papers should be carefully considered.

It is likely that regions with temperatures below 18°*C*, as is typical for the highland areas of East and Southern Africa, which are too cold for malaria transmission, might experience more malaria if their temperatures increase. However, malaria transmission in the future will be dependent on many other factors such as poverty, housing, access to medical care, host immunity and malaria control measures.

Most countries in Sub-Saharan Africa have annual mean temperatures between 20 and 28°*C*. In these areas, linking past and future temperature fluctuations to changes in malaria transmission is challenging. Our data suggest that one way to reduce this uncertainty is to use age-structured mosquito models. These models produce results that agree with the observed data, and non-exponential mosquito mortality has been demonstrated in several studies [33,40-42], although the true nature of mosquito survival in the field is not fully elucidated. The newly calibrated Martens 2 model described here also produces acceptable results. If simplicity is a goal in itself [43], models that assume exponential mortality will still have utility. To believe in projections of the potential impact of long-term, large-scale climate changes, it is crucial that models have an accurate representation of malaria transmission, even at the cost of complexity. For studies of malaria transmission at village level, other approaches might be more suitable [10,16,44,45].

## Abbreviations

BL: Bayoh and Lindsay; EIP: Extrinsic incubation period; ODEs: Ordinary differential equations.

## Competing interests

The authors declare that they have no competing interests.

## Authors’ contributions

The work presented here was carried out in collaboration between all of the authors. BL, MNB and TML defined the research theme. MNB provided the data for the control. TML designed the methods and experiments, did the model runs, analysed the data, interpreted the results, and wrote the paper. All authors read and approved the final version of the manuscript.

## Supplementary Material

Additional file 1 Details of the Bayoh-Lunde model, mosquito biting rate, and parasite extrinsic incubation period. Click here for file

Additional file 2 **This file shows how *ζ *can be used to change the shape of the Bayoh-Lunde survival curve.** The black line is the reference data, while the red line represents the Bayoh-Lunde survival curve. Temperature, relative humidity (as a fraction from 0 to 1), and *ζ* are given in the panel strips. Click here for file

Additional file 3 **Survival curves for all of the models investigated in this study plotted at different temperatures and relative humidities.** The figure on page two shows the legend as well as an example of non-exponential mortality. Click here for file

Additional file 4 A note on the use of ordinary differential equations, age structure (with an example), and rate calculations. Click here for file
