# Supplementary material for: How malaria models relate temperature to malaria transmission
Source: Parasit Vectors. 2013 Jan 18;6:20. doi: 10.1186/1756-3305-6-20 (PMC3598736; doi:10.1186/1756-3305-6-20)
Supplement: Additional file 1 — Details of the Bayoh-Lunde model, mosquito biting rate, and parasite extrinsic incubation period. [file 1756-3305-6-20-S1.pdf]

How malaria models relate temperature to malaria transmission: Details of the Bayoh-Lunde model, mosquito biting rate, and parasite extrinsic incubation period

Torleif Markussen Lunde*[1,2], Mohamed Nabie Bayoh[3] and Bernt Lindtjørn[2]

[1]Bjerknes Centre for Climate Research, University of Bergen, Norway
[2]Centre for International Health, University of Bergen, Norway
[3]KEMRI/CDC Research and Public Health Collaboration, Kisumu, Kenya

Email: Torleif Markussen Lunde*- torleif.lunde@cih.uib.no; Mohamed Nabie Bayoh - nbayoh@kemricdc.org; Bernt Lindtjørn - bernt.lindtjorn@cih.uib.no;

*Corresponding author

Bayoh-Lunde

From the Bayoh data set [1], Lunde *et al.* [2] derived an age-dependent mortality model that is dependent on temperature, relative humidity (RH), and mosquito size. In the subsequent text, we name this model Bayoh-Lunde. The four other models use daily survival probability as the measure, and assume that daily survival probability is independent of mosquito age. The model described here calculates a survival curve (ϖ) with respect to mosquito age. Like the Bayoh-Parham model, we also vary mortality according to temperature and RH.

Because mosquito size is also known to influence mortality [3–6], we have applied a simple linear correction term to account for this. The effect of mosquito size is minor compared with temperature and RH. In the presentation of our model, the first equation shows how the mortality rate can be calculated for a given age group. This is followed by the equation for the survival curve, while the last three equations show how mosquito size, RH, and temperature fit into the equation for the survival curve.

From the survival curve, we can calculate the probability that a mosquito will survive from one day to the next given its age. Equation 5 in the main manuscript shows the relationship between survival probability, p, and mortality rate, β, and the mortality rate can be translated to survival probability accordingly.

The mortality rate, $\beta_n(T, RH, size)$, in an age interval, n, is approximated as:

$$\beta_n(T, RH, size) = \left\{ \begin{array}{rl} \frac{\log\left(\frac{\varpi_{N,m_{t_2}}}{\varpi_{N,m_{t_1}}}\right)}{\Delta t} & \text{if } T < 40 \\ 3 & \text{otherwise} \end{array} \right\} \tag{1}$$

where the survival curve, $\varpi_{N,m}(\alpha, \zeta, a)$ (equation 2), is given as the fraction of live mosquitoes at day a (see additional files 1 and 3 for plots of the survival curves). The curve is designed such that during the first days of life, mortality will be low, followed by a stage of near exponential mortality. Mosquitoes surviving up to a given age will again have reduced mortality rates.

$$\varpi_{N,m}(\alpha, \zeta, a) = \sum_{i=0}^{a} \left(\frac{\left((\alpha \cdot a)^{\sum_{n=0}^{n=(\zeta-1)} n}\right)}{\sum_{n=0}^{n=(\zeta-1)} n!} \right) \cdot e^{(-\alpha \cdot a)} \tag{2}$$

where we subjectively set $\zeta = 6$, and α is given in equation 5.

The ζ parameter is used to control the shape of the survival curve in a similar manner as for the probability density functions. The figure in additional file 1 shows how ζ can be used to match the observed data. In the figure in additional file 3, we see how the functions behave when ζ is set to 6.

The α parameter is equivalent to a scale parameter in probability density functions. The smaller the value of α, the longer the survival. The α parameter is dependent on temperature, RH, and mosquito size.

These two parameters can be used to describe the impact of interventions such as insecticide-treated bed nets and indoor residual spraying, or as in this case, temperature and RH.

Mosquito size (defined as wing length in mm) goes into the equation as a linear correction factor. This correction factor, g, makes larger mosquitoes live longer than smaller ones. No correction is applied when the mosquitoes have a wing length of 3.05 mm (since $g(3.05) = 1$).

$$g(m_{size}) = 2.1731 - 0.3846 \cdot m_{size} \tag{3}$$

where m_{size_n} is mosquito size measured as mm wing length, and g goes into equation 5.

RH modulates α, the scale of the survival curve. Low RHs (starting from about 50%) will reduce survival. Between 60 and 100% the effect of RH is smaller.

$$f(RH) = 6.48007 + 0.69570 \cdot (1 - e^{-0.06 \cdot RH}) \tag{4}$$

where RH is given in % from $0 - 100$.

We now introduce information about mosquito size (g) and RH ($f(RH)$) to the scale parameter α, given in equation 5. At this stage, we also introduce information about temperature, which reduces the mosquito life span at low and high temperatures.

$$\alpha = g\left(m_{size}\right) \cdot e^{10+\left(1+\frac{T_{air}+1}{21}\right)^{(2/3)} \cdot \left(\left(1+\frac{(T_{air}+1)}{21}\right)^{2} - \left(1+\frac{T_{air}+1}{21}\right)\cdot 2 - f(RH)\right)} \tag{5}$$

g is a function of mosquito size, RH is relative humidity, and T_{air} is air temperature. In this exercise, we set mosquito size to ($m_{size_n} = 3[mm]$).

This is a more complicated expression, but as we shall see it also captures more of the dynamics of *Anopheles gambiae s.s.* mortality.

The mortality models have been implemented in the R-package, OMaWa, which includes examples of their usage, as well as their uses in malaria models that include mosquito hatching and the human population [7]. The survival curves used in this paper can be seen in additional file 3.

Biting rate and extrinsic incubation period

The equations used for the biting rate, $G(T)$, and the inverse of the extrinsic incubation period (EIP, pf) are described in Lunde *et al.* [2]. For convenience, we repeat them here.

The biting rate, $G(T)$, is assumed to be temperature dependent, while F_{gonot} is a weighting function described later. We also assume biting starts once the mosquito eggs have developed, and as such the biting rate is equal to the inverse of the length of the gonotrophic cycle (in days).

$$F_{gonot} = min\left(max\left(-\frac{2}{3} + \frac{1}{30} \cdot T_{air}, 0\right), .5\right) \tag{6}$$

$$G(T) = \left(1 + \frac{D_d}{T_{air} - T_c}\right)^{-1} \cdot F_{gonot} + \left(1.71 + 544347.6 \cdot T_{air}^{-3.93}\right)^{-1} \cdot \left(1 - F_{gonot}\right) \tag{7}$$

where T_{air} is in $^{\circ}C$. D_d is degree days, and T_c is the critical temperature from Hoshen and Morse [8], with $D_d = 37$, and $T_c = 7.7$. F_{gonot}, as defined in equation 6, is a weighting function; at higher temperatures the degree day concept by Hoshen and Morse [8] is used, while at lower temperatures more weight is given to a statistical relationship between temperature and the length of the gonotrophic cycle derived from Guillermo *et al.* [9, *An. albimanus*], Afrane *et al.* [10, *An. gambiae s.l.*], and Maharaj [11, *An. arabiensis*]. Since our main interest in this research was to examine how mosquito mortality was related to temperature in models, we used the same equation for the gonotrophic cycle in all of the mortality models. Had we used

different temperature dependent gonotrophic cycle estimates for the five models, we would not have been able to look at the effect of the mortality curves alone.

The development rate per day of sporozoites, pf, inside a mosquito can be written:

$$pf = \left(a + \frac{b}{e^{(T_{air})^c - d}}\right)^{-1} \tag{8}$$

where a = 9.5907, $b = 0.0051029$, $c = 0.7349$, and $d = 17.0325$.

This relationship was derived using data from MacDonald [12].

At lower temperatures, mosquito development will be slow, while at temperatures above $\approx 30°C$, the rate of development will remain constant (i.e., it does not increase with temperature). The model for pf does not contain any upper or lower temperature limitations. This can be justified because at low temperatures, the number of days required to develop sporozoites is more than an order of magnitude greater than the life span of the mosquitoes. At high temperatures, mosquito survival is so low that hardly any mosquitoes will live long enough to develop sporozoites, despite high rates of development.

References

1. Bayoh N: **Studies on the development and survival of Anopheles gambiae sensu stricto at various temperatures and relative humidities**. *PhD thesis*, University of Durham 2001.
2. Lunde TM, Korecha D, Loha E, Sorteberg A, Lindtjørn B: **A dynamic model of some malaria-transmitting anopheline mosquitoes of the Afrotropical region. I. Model description and sensitivity analysis [in press]**. *Malaria Journal* 2013.
3. Lyimo EO, Koella JC: **Relationship between body size of adult Anopheles gambiae s.l. and infection with the malaria parasite Plasmodium falciparum.** *Parasitology* 1992, **104 (Pt 2)**:233–7, [http://www.ncbi.nlm.nih.gov/pubmed/1594289].
4. Ameneshewa B, Service MW: **The relationship between female body size and survival rate of the malaria vector Anopheles arabiensis in Ethiopia.** *Med Vet Entomol* 1996, **10**(2):170–2, [http://www.ncbi.nlm.nih.gov/pubmed/8744710].
5. Fouet C, Gray E, Besansky NJ, Costantini C: **Adaptation to aridity in the malaria mosquito Anopheles gambiae: chromosomal inversion polymorphism and body size influence resistance to desiccation.** *PLoS One* 2012, **7**(4):e34841, [http://www.ncbi.nlm.nih.gov/pubmed/22514674].
6. Gray EM, Bradley TJ: **Physiology of desiccation resistance in Anopheles gambiae and Anopheles arabiensis**. *Am J Trop Med Hyg* 2005, **73**(3):553–559, [http://www.ajtmh.org/cgi/content/abstract/73/3/553].
7. **OMaWa**[ftp://ftp.uib.no/pub/gfi/tlu004/OMaWa/].
8. Hoshen M, Morse A: **A model structure for estimating malaria risk**. In *Environmental Change and Malaria Risk: Global and Local Implications*, *Volume 9*. Edited by Takken W, Martens P, Bogers RJ 2005:10.
9. Rua GL, Quinones ML, Velez ID, Zuluaga JS, Rojas W, Poveda G, Ruiz D: **Laboratory estimation of the effects of increasing temperatures on the duration of gonotrophic cycle of Anopheles albimanus (Diptera: Culicidae).** *Mem Inst Oswaldo Cruz* 2005, **100**(5):515–20, [http://www.ncbi.nlm.nih.gov/pubmed/16184229].
10. Afrane YA, Lawson BW, Githeko AK, Yan G: **Effects of microclimatic changes caused by land use and land cover on duration of gonotrophic cycles of Anopheles gambiae (Diptera: Culicidae) in western Kenya highlands.** *J Med Entomol* 2005, **42**(6):974–80, [http://www.ncbi.nlm.nih.gov/pubmed/16465737].

11. Maharaj R: **Life table characteristics of Anopheles arabiensis (Diptera: Culicidae) under simulated seasonal conditions.** *J Med Entomol* 2003, **40**(6):737–42, [http://www.ncbi.nlm.nih.gov/pubmed/14765646].

12. MacDonald G: *Dynamics of Tropical Disease*. Oxford University Press, London 1973.
