# Supplementary figures and images for: How malaria models relate temperature to malaria transmission
Source: Parasit Vectors. 2013 Jan 18;6:20. doi: 10.1186/1756-3305-6-20 (PMC3598736; doi:10.1186/1756-3305-6-20)

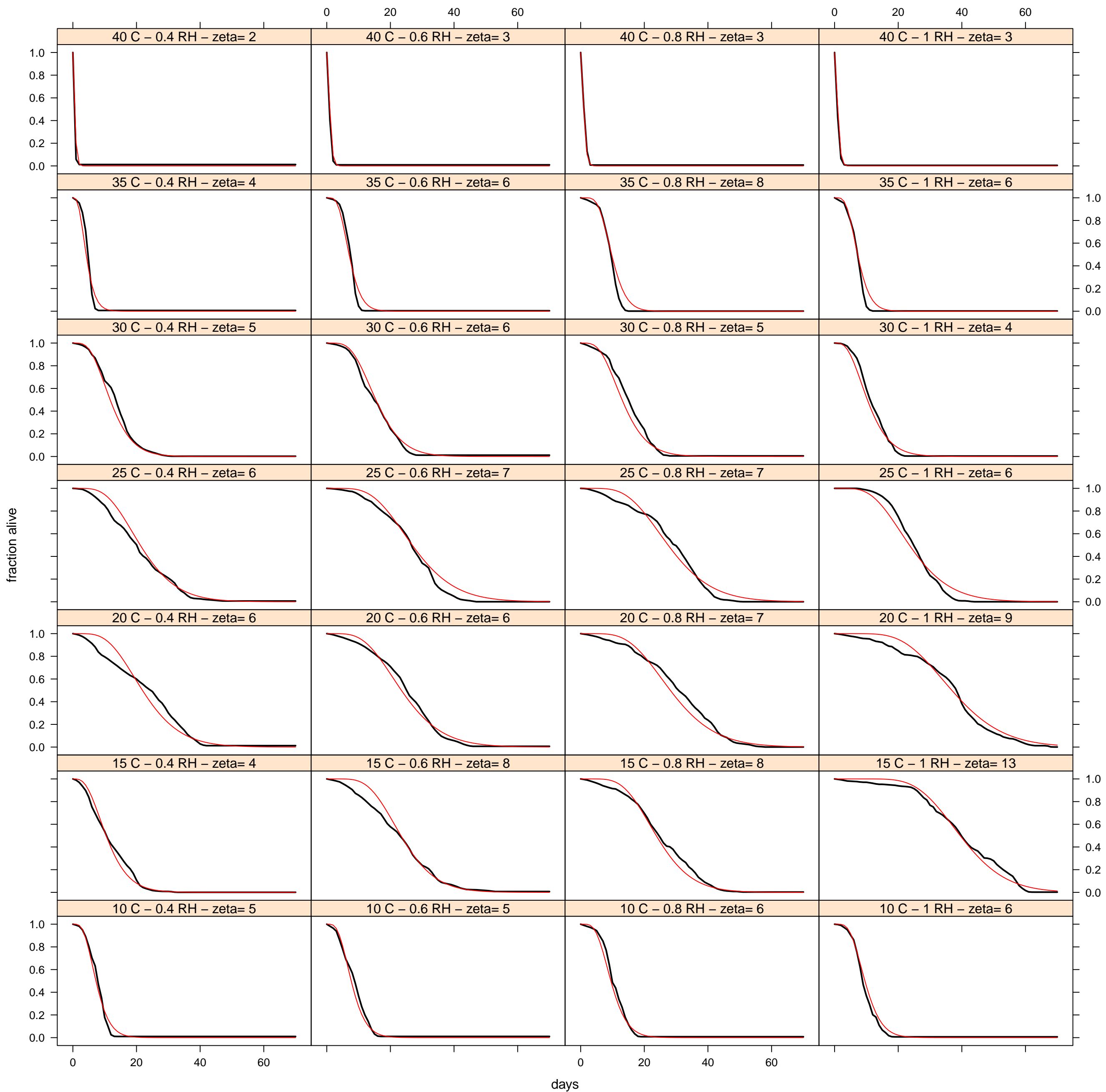

Supplement: Additional file 2 — This file shows how ζ can be used to change the shape of the Bayoh-Lunde survival curve. The black line is the reference data, while the red line represents the Bayoh-Lunde survival curve. Temperature, relative humidity (as a fraction from 0 to 1), and ζ are given in the panel strips. [file 1756-3305-6-20-S2.pdf]

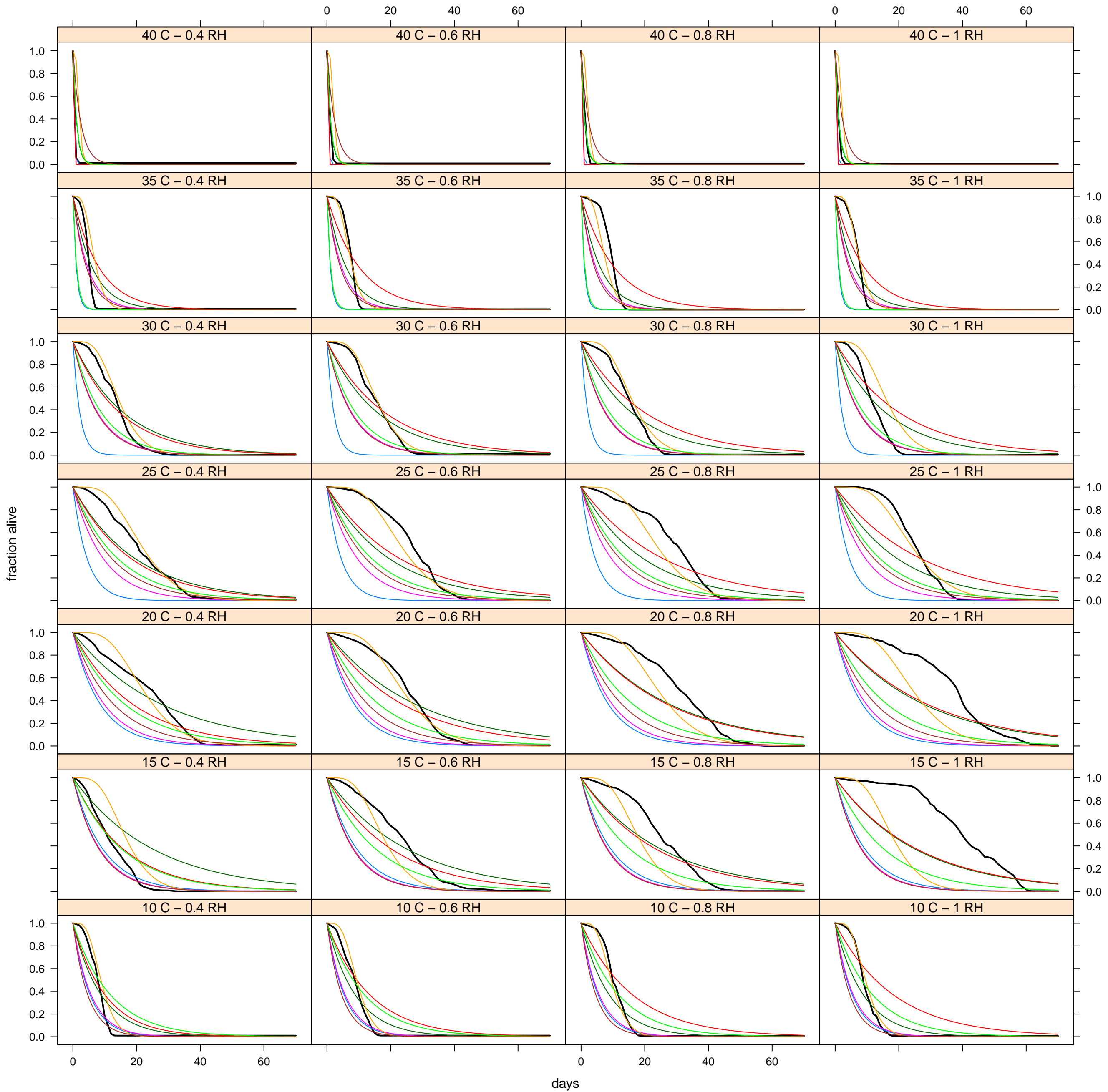

## Example of non-exponential mortality

25 C – 0.6 RH

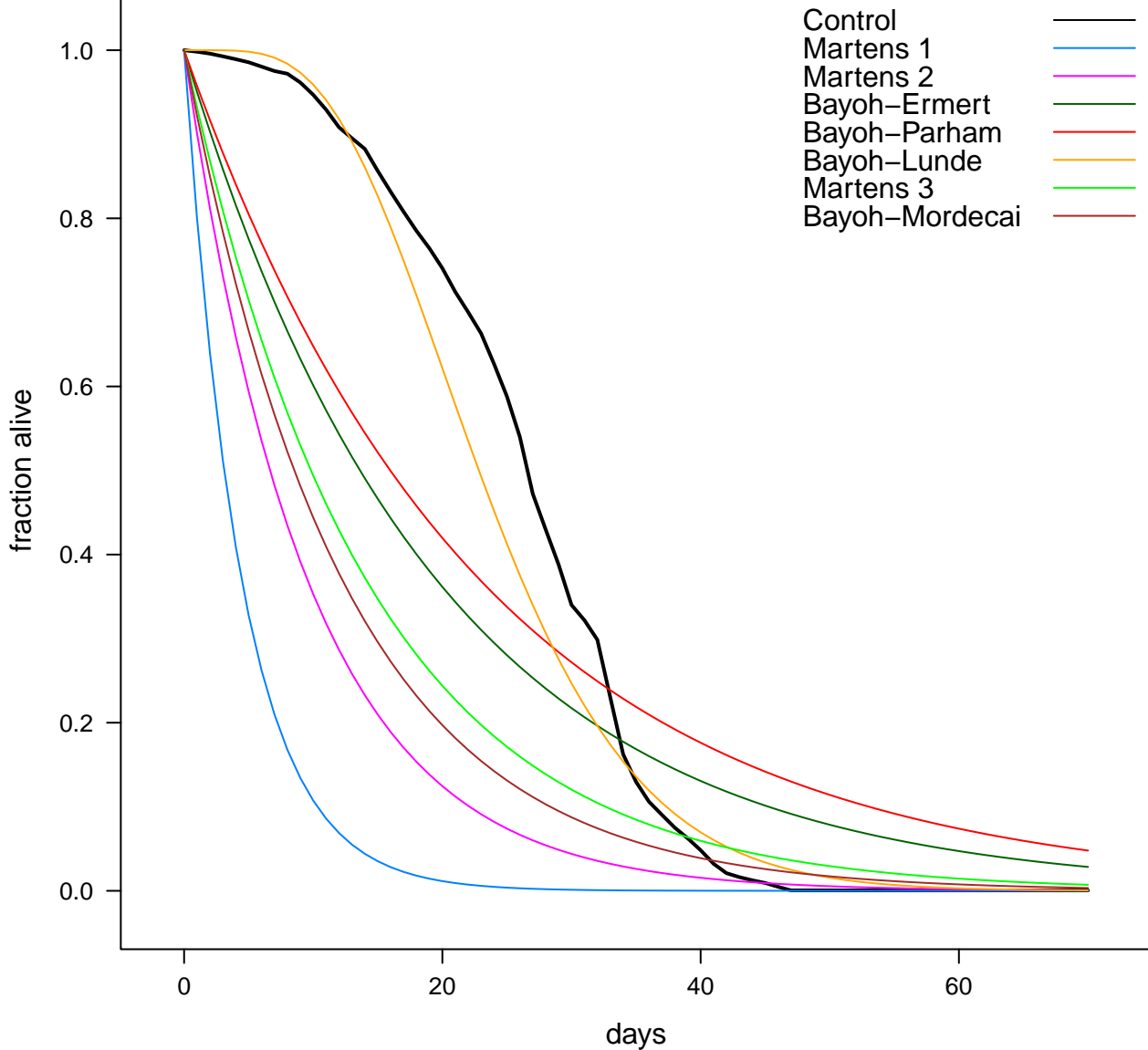

Supplement: Additional file 3 — Survival curves for all of the models investigated in this study plotted at different temperatures and relative humidities. The figure on page two shows the legend as well as an example of non-exponential mortality. [file 1756-3305-6-20-S3.pdf]
