# Supplementary material for: How malaria models relate temperature to malaria transmission
Source: Parasit Vectors. 2013 Jan 18;6:20. doi: 10.1186/1756-3305-6-20 (PMC3598736; doi:10.1186/1756-3305-6-20)
Supplement: Additional file 4 — A note on the use of ordinary differential equations, age structure (with an example), and rate calculations. [file 1756-3305-6-20-S4.pdf]

Torleif Markussen Lunde

November 20, 2012

1 Rates in ordinary differential equations

All models have biases. The simplest model is the correlation between two variables, where our interpretation decides if variable x is influencing variable y, or variable y is influencing variable x, or the two variables by coincidence vary in the same place. In a dynamical model, we write relationships as mathematical equations. As an example, we could use the development of a mosquito from pupa to adult. In real life, such a metamorphosis could be described by delay differential equations (equation 1), but for practical purposes they are often approximated and written as ordinary differential equations (ODEs, equation 2).

$$\begin{aligned} \frac{dP(t)}{dt} &= -P(t-\tau) \\ \frac{dA(t)}{dt} &= P(t-\tau) \end{aligned} \tag{1}$$

where τ is the number of days required to develop from pupa, P, to adult, A.

$$\begin{aligned} \frac{dP}{dt} &= -P \cdot r \\ \frac{dA}{dt} &= P \cdot r \end{aligned} \tag{2}$$

where A is the number of adults, P is the number of pupae, and r is the development rate from pupa to adult.

By deciding to use ODEs, we have introduced the first error. ODEs are capable of producing half a pupa and half a mosquito at any given time, and such pupae would converge towards zero. Let us continue the following example. We start with two pupae, $P = 2$, and zero adults $A = 0$, neglecting mortality. Development from pupa to adult takes 2 days. The exact solution of this problem would be
$P(t = 0) = 2, P(t = 1) = 2, P(t = 2) = 0$ and
$A(t = 0) = 0, A(t = 1) = 0, A(t = 2) = 2$.

In the framework of ODEs, the value of r would decide how fast development occurs. One method is to define the rate as per day, day^{-1}. In this case, $r = 1/2$. The exact solution using ODEs then becomes
$P(t = 0) = 2 \cdot e^{-r \cdot t} = 2.00, P(t = 1) = 1.21, P(t = 2) = 0.74$, and
$A(t = 0) = 2 - 2 \cdot e^{-r \cdot t} = 0, A(t = 1) = 0.79, A(t = 2) = 1.26$

Another way to define the development rate, r, is to consider the fraction of pupae that have developed at time t. Let us say that 50% of the pupae had developed into adults by the second day ($t = 1$); we could then find an exact solution that satisfies this condition:

$$
\begin{aligned}
&P(t=1) = P(t=0) \cdot e^{-r \cdot 1} = P(t=0) \cdot 0.5 \\
&e^{-r} = 0.5 \\
&r = -log(0.5)
\end{aligned} \tag{3}
$$

This approach then defines the development rate as the time it takes for 50% of the mosquitoes to develop from pupae to adults, $d(t)$, or more generally, $r = -log(0.5) \cdot d(t)^{-1}$.
